# Evolution of Multiple Additive Loci Caused Divergence between *Drosophila yakuba* and *D. santomea* in Wing Rowing during Male Courtship

**DOI:** 10.1371/journal.pone.0043888

**Published:** 2012-08-30

**Authors:** Jessica Cande, Peter Andolfatto, Benjamin Prud'homme, David L. Stern, Nicolas Gompel

**Affiliations:** 1 Institut de Biologie du Developpement de Marseille-Luminy, Aix-Marseille Université, Marseille, France; 2 Department of Ecology and Evolutionary Biology and the Lewis Sigler Institute for Integrative Biology, Princeton University, Princeton, New Jersey, United States of America; 3 Howard Hughes Medical Institute and Department of Ecology and Evolutionary Biology, Princeton University, Princeton, New Jersey, United States of America; University of Arkansas, United States of America

## Abstract

In *Drosophila*, male flies perform innate, stereotyped courtship behavior. This innate behavior evolves rapidly between fly species, and is likely to have contributed to reproductive isolation and species divergence. We currently understand little about the neurobiological and genetic mechanisms that contributed to the evolution of courtship behavior. Here we describe a novel behavioral difference between the two closely related species *D. yakuba* and *D. santomea*: the frequency of wing rowing during courtship. During courtship, *D. santomea* males repeatedly rotate their wing blades to face forward and then back (rowing), while *D. yakuba* males rarely row their wings. We found little intraspecific variation in the frequency of wing rowing for both species. We exploited multiplexed shotgun genotyping (MSG) to genotype two backcross populations with a single lane of Illumina sequencing. We performed quantitative trait locus (QTL) mapping using the ancestry information estimated by MSG and found that the species difference in wing rowing mapped to four or five genetically separable regions. We found no evidence that these loci display epistasis. The identified loci all act in the same direction and can account for most of the species difference.

## Introduction

Behavior, especially courtship behavior, tends to be among the most rapidly evolving characteristics of sexually-reproducing animals [1], [2]. While, over the past 20 years, we have significantly advanced our understanding of the genetic basis for morphological evolution [3]–[5], we currently have little understanding about how innate behavior has evolved. We have as yet no illustration of evolutionary genetic changes that have produced functional changes in neural circuitry resulting in species-level differences in behavior. We have only fragmentary evidence for the genetic basis for behavioral evolution between species, mostly based on QTL studies which give a lower bound on the number of genes controlling a specific behavior and describe their genetic interactions (for example, see [6]–[9]).

Innate behaviors can be understood not just at the level of the cellular neural circuitry, which produces them, but also at the level of the genes coding for the development and activity of that circuitry. The latter remain a premier entry point into a particular neural circuit controlling a specific behavior [10], [11], in particular when one wants to compare circuits between species with divergent behaviors. It is likely that the genes underlying a change in innate behaviors between closely related species have altered neural circuits without “breaking” them to generate novel behavioral outputs. We sought to identify a simple and defined system for the study of behavior evolution, and to find genetic entry points into the underlying neurobiology: we therefore surveyed courtship behaviors in fruit fly species, looking for quantifiable, highly penetrant, innate behavioral differences that we could exploit for genetic mapping.

Both technical and biological considerations make male courtship behavior in *Drosophila* species an appealing experimental system with which to bridge the gaps between neural networks, behavior and evolution. On the technical side, at least 12 fly genomes have been sequenced [12], a pipeline for rapid genetic mapping using high throughput methods has been established [13], and transgenic tools have been adapted for and shown to work in a number of fly species [14], [15]. Particularly within the *D. melanogaster* subgroup [16], a number of species pairs are known to have syntenic genomes [17] and to generate fertile hybrids [18], making this subgroup ripe for a genetic mapping approach [19].

Male courtship behavior consists of repeated performance of multiple behavioral elements that utilize all sensory modalities. Courtship behavior is largely innate and reproducible under fixed conditions [20]. In *D. melanogaster*, the male first orients towards the female, taps her with one of his forelegs, then follows and sings to her by extending and vibrating one wing. Song bouts are interspersed with genital licking, abdomen drumming on the substrate, abdomen curling (copulation attempts), and ultimately may result in copulation [20], [21]. This succession of elementary steps constitutes a backbone sequence generally recognizable across species, but one that has evolved rapidly through the gain, loss or modification of individual steps [22].

This evolutionary trend was evident when we surveyed male courtship behavior in the *D. melanogaster* species subgroup. We focused on one particularly striking step, a slow rotational movement of the wing termed the “wing row”, present in 7 out of the 9 species in the *D. melanogaster* species subgroup [23]. Here we describe and map wing rowing differences between the sister species *D. yakuba* and *D. santomea* [24]. *D. yakuba* and *D. santomea* diverged approximately 400,000 years ago [25]. The two species occupy different habitats, and differ in morphological characteristics such as adult pigmentation [26], [27], genital morphology [24] and cuticular hydrocarbons [28]. While male courtship between the two species is markedly similar, it differs in several key respects, such as the parameters of their courtship song [29] and wing rowing. In spite of an overlap in their geographical ranges on the island of São Tomé, hybrids are found rarely in the wild, and a number of pre- and post- zygotic isolating mechanisms between the two species have been described in the lab, including mate choice discrimination and F1 hybrid male sterility [24], [30]. Using multiplex shotgun genotyping (MSG), we performed quantitative trait locus (QTL) mapping on wing rowing frequency and localized the evolved loci to four or five QTL, which are sufficient to account for at least 70% of the difference in this trait between these two species.

## Results

### Courtship and wing rowing in the *melanogaster* subgroup

**Figure 1:**
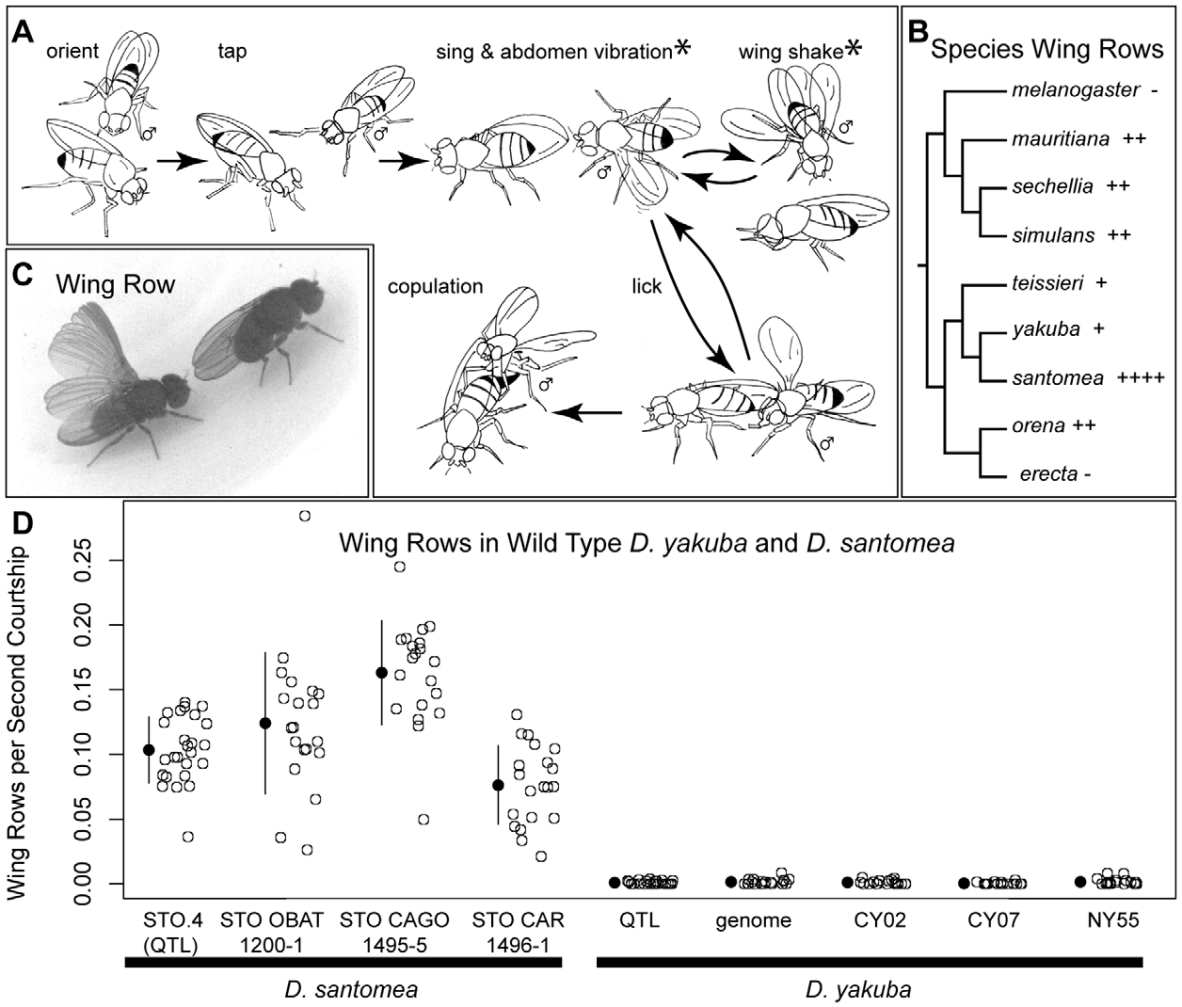
Courtship behavior in wild type *D. yakuba*and *D. santomea*. (A) Males of both species first orient towards the female and tap her with a T1 leg. Then they approach the back or the side of the female and periodically sing by vibrating one extended wing. Song bouts are punctuated by circling to the side and front of the female (circling is sometimes accompanied by shaking of both wings) or by attempts to lick the female's genitalia. If the female is receptive, a lick is followed immediately by copulation. Cartoons were adapted from movie still images. Courtship steps at which *D. santomea* males are observed to row are marked with an asterisk (*). (B) Distribution of wing rowing in the species of the *D. melanogaster* species subgroup. The molecular phylogeny was adapted from Prud'homme et al. [55]. (−) and (+) indicate which species row, and relative rowing frequencies (rows/second courtship): *D. mauritiana* = 0.0322; *D. sechellia* = 0.0228; *D. simulans* = 0.0279; *D. teissieri* = 0.0061; *D. yakuba* = 0.0010; *D. santomea* = 0.1033; *D. orena* = 0.0144. (C) Chronophotograph of a *D. santomea* male rowing while positioned behind a stationary female. (D) The frequency of rowing in multiple independent *D. santomea* and *D. yakuba* isolates. y-axis: wing rows normalized to seconds of courtship in a 15 minute movie. Sample means are marked by the filled circle and lines indicate +/− one standard deviation. Species level differences *D. santomea* and *D. yakuba* lines were highly significant (Nested Anova; D.F. = 4,184; F = 266.4; p<2.2e-16), while only *D. santomea* STO CAGO 1495-5 rowed significantly more than the other *D. santomea* isolates (Anova; D.F. = 3, 81; F = 18.26; p<1.62e-06) and there were no significant differences between *D. yakuba* isolates.

We surveyed courtship behavior in *Drosophila* species and looked for qualitative and quantitative differences in species known to form viable hybrids, and for which a genome was available for at least one of the parent species [12]. Courtship behavior in the *D. melanogaster* species subgroup follows a readily recognizable pattern: males initially orient towards the female, tap her, and then give chase. This is followed by bouts of unilateral wing extension and vibration in all species (singing), interspersed with a suite of other wing movements (e.g. scissors, flicks and rowing), abdomen movements such as bobbing and curling, and circling and display behaviors, many of which vary from species to species in a stereotypical fashion [22], [31], [32]. A cartoon of male courtship for *D. yakuba* and *D. santomea* (Fig. 1A) captures this characteristic pattern of the subgroup. Unlike the slower abdomen drumming seen in *D. melanogaster* [21], *D. yakuba* and *D. santomea* males will also simultaneously rapidly vibrate their abdomens while extending their wings (visible in Movie S1). In both *D. yakuba* and *D. santomea*, a display behavior punctuates song bouts, wherein the male shakes both wings while slowly circling the female (Movie S2). Males of all species will periodically try to lick female genitalia, and ultimately a lick is followed by copulation [32].

Most species in the subgroup perform a distinctive wing movement, the wing row. Seven of the nine species of the *D. melanogaster* species subgroup row during courtship (Fig. 1B, representative rowing clips in Movie S3) [23], [31], [32]. During rowing, the male extends and rotates the wing so that the wing blade faces the female before returning it to a position parallel to the substrate (depicted in a chronophotograph of a *D. santomea* male in Fig. 1C). This behavior varies qualitatively and quantitatively between species. First, some species tend to row only one wing at a time (e.g. *D. teissieri*), while others usually row both wings (e.g. *D. sechellia*) and some, like *D. santomea*, do both types of behaviors (Movie S3) [31]. The phylogenetic distribution of rowers versus non-rowers (Fig. 1B) suggests that wing rowing is ancestral to this clade, and has been lost separately both in *D. melanogaster* and in *D. erecta*. We observed a rare slow wing extension in *D. erecta*, which has not been described previously [32] (Movie S3). This may or may not be related to rowing behavior. Nevertheless, true rowing seems to be highly reduced, if not absent, in *D. melanogaster* and *D. erecta*. Second, among rowing species, the frequency of rowing varies amongst species (Fig. 1B).

We focused on *D. yakuba* and *D. santomea*, a hybridizing species pair that spans the gamut of rowing behaviors: *D. yakuba* almost never rows, while *D. santomea* rows an order of magnitude more frequently than any other species in the *D. melanogaster* subgroup (Fig. 1B and 1D). In *D. santomea*, but rarely in *D. yakuba*, bouts of courtship song, as well as wing shake and circling displays are punctuated by wing rowing. Rowing is sometimes limited to one wing and sometimes involves both wings in rapid sequence (starred courtship steps in Fig. 1A, chronophotograph of a unilateral wing row shown in Fig. 1C, Movies S1, S2, S3, S4). Males of both species row only when stationary. To ascertain the stability of this behavioral shift in wing rowing frequency in *D. yakuba* and *D. santomea*, we quantified the frequency of rowing in four *D. santomea* and five *D. yakuba* independent lines (Fig. 1D). In brief, pairs of courting flies were filmed for 15 minutes and the total number of rowing events was counted and normalized to the total time spent courting. *D. santomea* males rowed, on average, once every 8.6 seconds. Only one of the *D. santomea* lines (STO CAGO 1495-5) rowed significantly more than the others (Fig. 1D). In contrast, there were no statistically significant differences between the 5 independent *D. yakuba* lines, and *D. yakuba* males rowed, on average, only once every 100 seconds (Fig. 1D). Thus, the frequency of wing rowing differs by over an order of magnitude between the two species, and this species level difference is stable across multiple independent lines, making variation in wing rowing frequency a viable candidate behavior for genetic mapping in *D. yakuba* and *D. santomea*.

### Wing rowing in *D. yakuba* and *D. santomea* hybrid crosses

**Figure 2:**
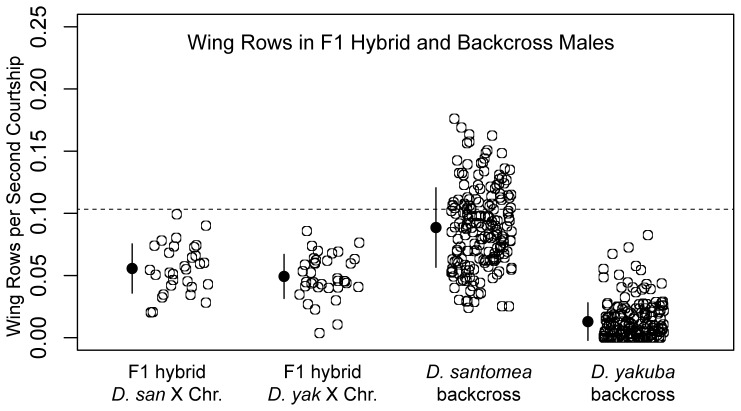
Wing rowing in F1 and backcross hybrid males. y-axis: wing rows normalized to seconds courtship observed in a 15 minute movie. x-axis from left to right: F1 hybrid males from *D. santomea* females crossed to *D. yakuba* males; F1 hybrid males from the reciprocal cross; males from an F1 hybrid female backcrossed to a *D. santomea* male; males from an F1 hybrid female backcrossed to a *D. yakuba* male. Levels of wing rowing in F1 hybrid males from either cross direction are not significantly different (Student's t-Test; t = 1.3672; D.F. = 58.86; p = 0.1768; two-tailed) indicating there are no contributing loci on the X chromosome. Filled circles represent the mean level of rowing and lines +/− one standard deviation. Dashed line indicates mean wing rowing frequency for the *D. santomea* parental line from Fig. 1.

To elucidate the genetics of wing rowing variation in these two species, we quantified rowing in F1 hybrid males and in backcross offspring produced by crosses in both directions between *D. santomea* and *D. yakuba*. On average, rowing in F1 hybrid males was intermediate between the two parent species, indicating that wing rowing is not controlled by a single dominant or recessive locus. In addition, the distributions of wing rowing behavior did not differ between F1 progeny resulting from reciprocal parental crosses, which suggests that there are no additive loci of large effect on the X chromosome that contribute to this behavior (Fig. 2).

The backcross progeny displayed rowing frequencies that were, on average, intermediate between the rowing frequencies of the relevant parental lines and the F1 hybrids (Fig. 2). Very few backcross individuals displayed rowing phenotypes that were significantly more extreme than the parental *D. santomea* distribution (Fig. 2), and *D. yakuba* backcross individuals that rowed generally did so at a frequency less than that of the *D. santomea* parental line, or even the F1 hybrids. This suggests that there is little or no transgressive segregation [33] in these crosses. This pattern of backcross segregation for the wing rowing phenotype indicates that the difference between the two species is controlled by multiple autosomal loci.

### Quantitative trait locus mapping

We employed Multiplexed Shotgun Genotyping (MSG) [13] to estimate the ancestry of chromosome regions for use in quantitative trait locus (QTL) mapping. DNA isolated from multiple individuals was barcoded and pooled into a single sequencing library (File S1). Using MSG software, the data from each individual were mapped to each of the two parental genomes and the allelic differences were used to estimate ancestry of chromosome regions with a Hidden Markov model (HMM) (Files S2 and S3).

MSG requires estimates of the parental genomes. We therefore generated genome sequences for the parental lines used in the crosses by updating the published *D. yakuba* genome with ∼116 million and ∼112 million filtered 100 bp Illumina reads for the *D. yakuba* and *D. santomea* parents, respectively. These reads were mapped to the published *D. yakuba* genome [12] and approximately 74.2% and 69.2% of the genome was updated with *D. yakuba* and *D. santomea* reads, respectively. Using ancestry estimates from MSG, we determined linkage relationships between contiguous markers and found two regions of the published *D. yakuba* genome that appear to be misassembled based on linkage information. On chromosome 2 L, one region of ∼2.5 Mb was inverted in place and, on chromosome 2R, one region of ∼2.4 Mb was inverted and displaced from its correct location by ∼7 Mbp (Fig. S1 & Table S1). Multiple small regions of chromosome 2, 3 and X did not show strong linkage to other markers in the genome. We generated new parental genomes by inverting and repositioning the two inverted regions and by masking the remaining small regions that showed unusual linkage patterns. We then re-ran the entire analysis with these rearranged genomes.

### Marker generation with MSG

**Figure 3:**
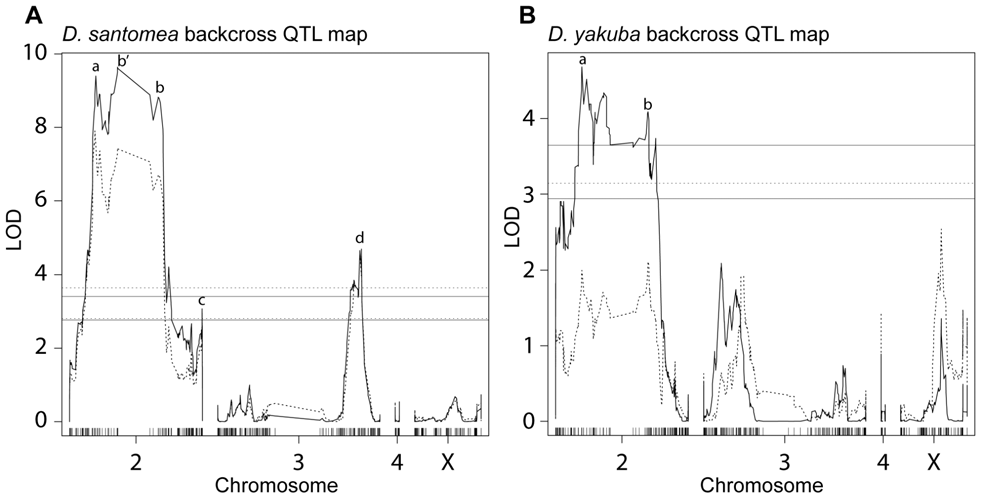
QTL analysis of wing rowing in *D. santomea* and *D. yakuba* backcross males. QTL maps of the *D. santomea* backcross (A) and *D. yakuba* backcross (B) flies from Fig. 2. LOD is indicated on the y-axis. The x-axis is the physical map based on the *D. yakuba* genome, SNP markers are represented as black tick marks. Solid lines represent the LOD scores for wing rows normalized to seconds courtship, dashed lines for normalization to seconds the female was stationary during courtship (WWPSSF). Solid and dashed horizontal lines illustrate the corresponding permutation-determined 0.05 (lower) and 0.01 (upper) confidence limits, respectively. The 0.01 confidence limit in the *D. yakuba* backcross for stationary female normalization equals 5.02 and is not shown in panel B. In (A), a, b′, b, c and d indicate QTL at positions chr2:8,591,051, chr2:15,687,032, chr2:30,610,169, chr2:43,343,589 and chr3:46,460,168, respectively. In (B), a and b indicate QTL at chr2:8,591,087 and chr2:32,778,893.

MSG software estimates conditional probabilities of ancestry given the data, while accounting for multiple sources of error [13]. Ancestry was estimated at 150,808 and 143,051 genomic locations (about one marker per kb, on average) for the *D. santomea* and *D. yakuba* backcrosses, respectively, but most neighboring markers encoded redundant information. We therefore thinned the data to neighboring markers whose conditional probabilities differed in at least one individual by at least 0.1. This thinning resulted in 1,720 and 1,713 markers for the *D. santomea* and *D. yakuba* backcrosses, respectively (Files S4 and S5). This represents about one marker per 100 kb on average. However, markers are not distributed evenly, or randomly, along the chromosome. Instead, the thinned, informative markers are over-represented close to recombination breakpoints and under-represented in regions of low recombination. This variable marker density can be observed in the “rug” of markers illustrated along the x-axis of Figs. 3A and B. Missing ancestry estimates, which occur at the ends of most chromosomes in most individuals, were converted to the prior probability of ancestry in a backcross, or 0.5. These conditional probabilities were then imported into R/qtl and used directly (i.e. conditional probabilities were *not* estimated from “hard” genotype calls) in Haley-Knott regression [34].

### QTL analysis of the *D. santomea* backcross

A genome scan for single, additive QTL for wing rows per second of courtship revealed a broad, and highly significant, QTL plateau stretching from ∼8.6 Mb to 30 Mb on chromosome 2, a weakly significant QTL peak at ∼43 Mb on chromosome 2, and a significant QTL peak at ∼47 Mb on chromosome 3 (solid lines in QTL profile of Fig. 3A). Since male flies never row while they are chasing females, we also scored wing rowing by excluding periods of courtship during which the female was running to escape the male (decamping) [22], [32]: wing rows per second stationary female (WRPSSF). We observed a LOD profile for WRPSSF (dashed lines in QTL profile of Fig. 3A) that was similar to, but weaker than, the LOD profile for wing rows per second of courtship.

The width of the large QTL plateau on chromosome 2 may be explained, partly, by an ∼10 Mb region of very low recombination that spans the centromere (Fig. 3A, spanning a, b′ and b). This region can be observed as a stretch of the middle of chromosome 2 that is devoid of markers. This paucity of markers results from the thinning procedure to remove most redundant markers and not from an absence of markers in the original data. However, the centromeric region of low recombination does not fully explain the width of this plateau, because the plateau extends into regions of substantial recombination. We therefore tested for the existence of multiple QTL in this region using multiple-QTL models [35]. A model with three QTL on chromosome 2 and a single QTL on chromosome 3 at ∼46.5 Mb provided a substantially better fit to the data than models with fewer QTL on chromosome 2 and provided a nearly equivalent fit to a model with more QTL (Table 1). The four QTL model with the highest LOD included QTL on chromosome 2 at ∼8.6 Mb, ∼29 Mb, and ∼43 Mb (Fig. 3A, QTL a, b, c and d). However, this model has only a marginally higher LOD score than a model with QTL on chromosome 2 at ∼8.6 Mb, ∼15.7 Mb, and ∼43 Mb (Fig. 3A, QTL a, b′, c and d). The best-fit model does not include a QTL at ∼15.7 Mb (Fig. 3A, QTL b′), the location with the highest LOD score in the single-QTL scan. This peak may represent an artifact of the single QTL scan, which can incorrectly imply the existence of a stronger QTL, a “ghost QTL”, between two linked QTL [36], although there is insufficient evidence to exclude the model containing the second chromosome 2 QTL at position ∼15.7 Mb.

**Table 1:** Best fit QTL models for different numbers of QTL for the *D. santomea* and *D. yakuba* backcrosses.

Backcross Direction	Number of QTL in Model	LOD of model	Percent Variance Explained	QTL locations	Effect Sizes†	LOD Drop One‡
*D. santomea*	2	13.89	28.33	chr2:15,687,032	−0.028	9.24
				chr3:46,460,168	−0.018	4.25
*D. santomea*	3	17.09	33.63	chr2:15,687,032	−0.0268	9.28
				chr2:43,343,589	−0.0158	3.2
				chr3:46,460,168	−0.0177	4.42
*D. santomea*	4	18.97	36.56	chr2:8,591,051	−0.0153	2.35
				chr2:28,967,599	−0.0177	3.2
				chr2:43,338,544	−0.0148	2.91
				chr3:46,460,168	−0.0167	3.97
*D. santomea*	§	18.84	36.36	chr2:8,591,051	−0.0139	1.75
				chr2:15,687,032	−0.0184	3.07
				chr2:43,343,589	−0.0157	3.3
				chr3:46,460,168	−0.0157	3.56
*D. santomea*	5	18.93	36.49	chr2:8,591,051	−0.014	1.76
				chr2:15,687,032	−0.015	0.76
				chr2:30,610,169	−0.005	0.09
				chr2:43,343,589	−0.016	3.18
				chr3:46,460,168	−0.016	3.63
*D. yakuba*	**	6.19	13.8	chr2:8,591,087	0.0078	2.45
				chr2:32,778,893	0.0061	1.51
*D. yakuba*	3	5.82	13.02	chr2:8,591,087	0.0068	1.19
				chr2:15,756,813	0.0009	0.01
				chr2:30,264,951	0.0051	0.38

† For the *D. santomea* backcross, this is the effect of introducing one *D. yakuba* allele of the specified QTL, and vice versa for the *D. yakuba* backcross.

‡ Log likelihood ratios comparing the full model to a model with the specified QTL removed.

§ This alternative four QTL model for the *D. santomea* backcross did not have a substantially lower LOD score than the best-fit four QTL model, suggesting that it is not possible to differentiate between a QTL at ∼15.7 Mb and at ∼29 Mb on chromosome 2.

** An alternative two QTL models with one QTL at ∼15.7 had LOD scores at least 0.76 LOD lower than the best-fit model.

We found no evidence for epistasis amongst QTL for wing rowing in the *D. santomea* backcross. We tested both for epistasis amongst the four QTL in the best fit model and we performed genome-wide pairwise tests amongst all markers for non-additive interactions. We found no significant LOD scores for any pairs of markers (Table S2).

**Figure 4:**
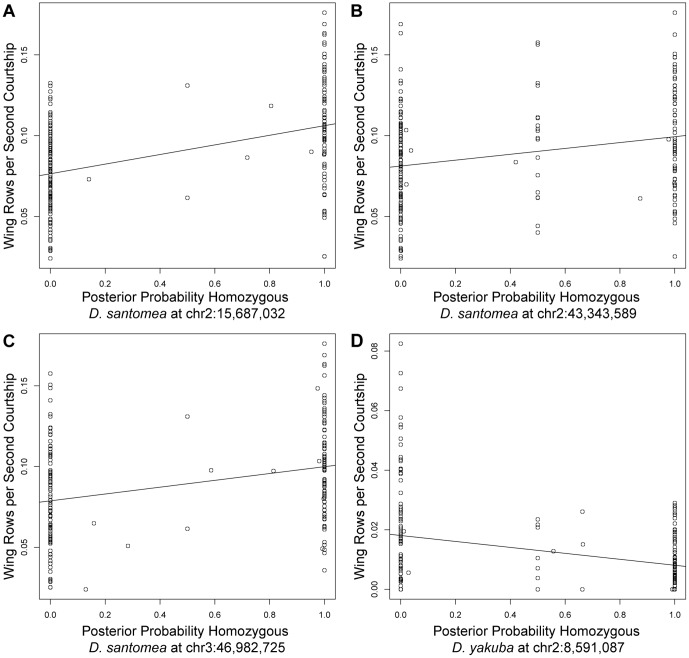
Effect plots for wing rowing QTL. (A) Effect plot for the QTL on chromosome 2 at 15,687,032 bp for the *D. santomea* backcross. (B) Effect plot for the QTL on chromosome 2 at 43,343,589 bp for the *D. santomea* backcross. (C) Effect plot for the QTL on chromosome 3 at 46,982,725 bp for the *D. santomea* backcross. (D) Effect plot for the QTL on chromosome 2 at 8,591,087 bp for the *D. yakuba* backcross. For each plot, the posterior probability of homozygosity as predicted by the HMM for backcross hybrid flies at the marker for the peak LOD score for each QTL is plotted on the x-axis. Heterozygous flies have a value of 0, homozygous flies a value of 1, and flies where the genotype is uncertain due to low marker density fall between zero and one. The number of wing rows normalized to seconds spent courting is plotted on the y axis.

The four QTL in the best-fit multiple QTL model explain ∼36% of the total phenotypic variance segregating in the backcross offspring. The remaining variance amongst the backcross progeny probably results mainly from uncontrolled environmental factors, such as female behavior, and additional loci of smaller effect. The four QTL in the best fit model have similar effect sizes and all *D. yakuba* alleles act in the same direction, to reduce the frequency of wing rowing (Table 1 and Fig. 4A–C). Assuming complete additivity, the estimated combined effect of a single *D. yakuba* allele at all four loci in a *D. santomea* background decreases rowing frequency by 0.065, which is equal to approximately 60% of the species difference (Table 1).

### QTL analysis of the *D. yakuba* backcross

In the *D. yakuba* backcross, a genome scan for single, additive QTL revealed a broad QTL plateau stretching from ∼8.6 Mb to 30 Mb on chromosome 2, in the same approximate location as the QTL plateau found in the *D. santomea* backcross. No other significant additive effects were detected elsewhere in the genome. A model with two QTL on chromosome 2, at ∼8.6 Mb and ∼32 Mb, provided a substantially better fit than a single QTL or a three QTL model (Table 1 and Fig. 3B, QTL a and b). This model explained 13.8% of the phenotypic variance in backcross offspring. The combined effect of these two *D. santomea* alleles in a *D. yakuba* backround increases rowing frequency by approximately 0.014, which equals approximately 13% of the species difference (Table 1, Fig. 4D).

The best locations for the two QTL found in the *D. yakuba* backross (∼8.6 Mb and 30 Mb, Fig. 3B QTL a and b) are located close to the best positions for two of the QTL found in the *D. santomea* backcross (∼8.6 Mb and 29 Mb, Fig. 3A QTL a and b). It is possible that these QTL represent semi-dominant effects of the same alleles acting in both backcross directions. We did not detect QTL near 43 Mb on chromosome 2 nor near 46 Mb on chromosome 3 in the *D. yakuba* backcross, but this may be because these QTL in the *D. santomea* backcross have smaller-magnitude effects than the QTL near 8.6 and 32 Mb on chromosome 2 in the *D. santomea* backround (Fig. 3). We did not detect any significant epistatic interactions in the *D. yakuba* backcross between the two QTL on chromosome 2 nor between any pairwise markers tested genomewide.

## Discussion

Our analysis establishes that the difference in wing rowing between *D. yakuba* and *D. santomea* is a polygenic trait that is determined mainly by variation at a handful of additive loci. We uncovered 4–5 QTL in the *D. santomea* backcross direction, and 2 QTL in the *D. yakuba* backcross direction. Several QTL are clustered on chromosome two from 8–28 Mbp. The region from 8–28 Mbp spans the centromere and we recovered few recombinants with breakpoints in this region. It is not clear if low recombination in this region is a result of the interspecies cross or if *D. santomea* and/or *D. yakuba* show reduced levels of recombination in this region. This reduced recombination is not due to an inversion between the two species, as no inversions were visible in polytene chromosome spreads, and recombination in this region is reduced but not absent. Resolution of this issue awaits further genetic studies of these species. In any case, this local low recombination reduces the resolution of QTL, as shown by our inability to distinguish statistically between alternative four-QTL models and between the four- and five-QTL models. Resolution of the QTL of this genomic stretch will require more targeted genetic approaches.

In the best fit four-QTL model for the *D. santomea* backcross, each QTL accounts for, on average, roughly 10–20% of the difference in wing rowing frequency seen between the parent species. While there may be additional loci contributing to variation in wing rowing, whose effects are too small to be detected in this analysis, the additive QTL identified here control the majority (at least 70%) of the variation in wing rowing between the two species.

One of the chromosome 2 QTL in the *D. santomea* backcross, at ∼29 Mb, lies within 4 Mb of one of the two QTL in the *D. yakuba* backcross best fit model. A second *D. santomea* backcross QTL, at ∼8.6 Mb, lies virtually on top of the other *D. yakuba* backcross QTL. If these two pairs of QTL represent the same two semi-dominant loci mapped in either direction, then wing rowing frequency in *D. yakuba* and *D. santomea* could depend on as few as four loci. These results provide a minimum estimate of the number of genes underlying wing rowing frequency. However, previous QTL studies have shown that, upon further analysis, large effect QTL such as these can decompose into QTL with smaller effect and that may interact epistatically (e.g. [37]). Finally, the effects of these QTL seem purely additive, as we found no evidence for epistatic interactions between any of the QTL.

There are several possible reasons for why only two QTL are detected in the *D. yakuba* backcross and for why they have smaller effect sizes than the QTL in the *D. santomea* backcross. The *D. santomea* alleles, on their own or in combination, may have smaller effects in a largely *D. yakuba* genetic background than they do in a *D. santomea* genetic background. In addition, given the observed phenotypic variance in the backcross progeny, we had limited power to detect QTL with effect sizes smaller than the two QTL found in the *D. yakuba* backcross. Similarly, the non-normal distribution of the *D. yakuba* backcross phenotype data may have had an effect, although standard interval mapping still generally works well if significance is established with a permutation test [35]. Finally, one or more of the *D. santomea* alleles that promote wing rowing may be insufficient, on their own, to induce wing rowing in the heterozygous state and our sample size may be too small to detect this epistasis.

There is an intriguing similarity amongst the results of insect behavior QTL studies over the last decade. Interspecies differences in courtship song have been mapped in the fruit flies *D. pseudoobscura* and *D. persimilis* [38], in *D. simulans* and *D. sechellia* [9], as well as in the hawaiian cricket *Laupala* [39]. In all cases, variation in song, like wing rowing, maps to a handful of loci (3–6), with no marked concentration of loci on the X chromosome. Similar results have been obtained when looking at mating success in fruit flies [40], [41], foraging behavior in honey bees [42], and at intraspecific variation in *D. melanogaster* aggression [43]. This may be an artifact, in that traits with many QTL of small effect will be difficult to map, or this may represent a real trend in the genetic architecture underlying behavioral traits in insects.

The physical linkage of functionally related genes—such as those involved in signals and signal recognition [39] or the localization of genes important for speciation to regions of low recombination such as inversions [38], [44], [45], [46]—has been proposed to contribute to the propagation of complex behavioral differences within a population. For example, courtship behaviors critical to assortative mating in stickleback fish appear to be concentrated on the X chromosome [47], while QTL for color and color preference have been shown to be linked to one another in butterflies [8]. Moehring et al. looked at QTL controlling pre-zygotic [41] and post-zygotic [30] mating isolation in *D. yakuba* and *D. santomea*. Only two of the six described pre-zygotic QTL mapped near a QTL described in this study, and one of those was within the recombination desert on chromosome 2 [41]. Likewise, the only post-zygotic QTL to overlap the wing rowing QTL also mapped to the recombination desert on chromosome 2 [30]. QTL for inter- and intra- specific variation in courtship song rarely overlapped with the wing row QTL from this study [9], [38], [46], [48], [49], nor did QTL for cuticular hydrocarbons important for speciation in *D. simulans* and *D. sechellia* [50]. Given the lack of resolution in these prior studies, whether or not the QTL controlling wing rowing are linked to additional QTL that influence behavior or reproductive isolation awaits further analysis.

In the post-genomic, high-throughput sequencing era, identification of causal evolutionary variants is limited not by the development and scoring of genetic markers, but instead by our ability to score the phenotype reliably in large numbers of individuals that carry useful recombination events. Using MSG [13], we genotyped 384 flies at 1700 markers in a matter of weeks. The two main limitations to our study are (1) the cumbersome manual phenotyping pipeline involving hundreds of hours of video analysis, which we hope to automate in future studies, and (2) limited recombination, which is an inherent biological property of these species which we cannot change. Small sample sizes have a tendency to exagerate the effect size of any one QTL (the Beavis effect) [51], and the same is true for QTL found in regions of low recombination, such as those found near the centromere or telomeres [52]. Nevertheless, independently verifying QTL and identifying individual genes will depend on working around the barrier set by the low recombination frequency seen in some regions of fly genomes. One possibility is to employ selective phenotyping, for instance by using *D. santomea* and *D. yakuba* transgenic lines carrying a fluorescent (dominant) marker [14] to select for recombinants in a region of interest and thereby restrict further mapping efforts to informative genomic regions [53]. Using mapped transgenic markers [13], it may also be possible to introgress regions of the genome underlying QTL from one species into the other [27], and thus refine the genomic regions defined by this QTL study.

## Methods

### Fly stocks

*D. santomea* STO OBAT 1200-1, STO CAGO 1495-5 and STO CAR 1496-1 were originally provided by Manyaun Long [54]. *D. yakuba* CY02 and CY07 were collected in Nguti, Cameroon in 2002 [54] while NY55 was collected in Nairobi, Kenya in 2006 by P. Andolfatto, and the *D. yakuba* genome strain is available from the San Diego species stock center (#14021-0261.01). *D. sechellia* (14021-0248.25), *D. teissieri* (14021-0257.00) and *D. erecta* (14021-0224.01) were obtained from the San Diego Species Stock Center, while *D. mauritiana* (C164.1) was the gift of John Roote and was collected originally in Mauritius, Rivière Noire in 1973 by F. Lemeunier. *D. orena* was a gift from Jean David. *D. simulans* T8 was collected in Tanzania and came from Marie-Louise Cariou. All flies were reared on cornmeal-agar media according to standard methods [18].

*D. santomea* STO.4 (San Diego Species Stock Center # 14021-0271.00) and the *D. yakuba* line #14021-0261.00 were used for QTL analysis. *D. yakuba* virgin females were crossed en masse to *D. santomea* males to generate F1 hybrid females, which were subsequently backcrossed to either parent line. Single *D. yakuba* backcross males were placed with single *D. yakuba* females from the parental line, and *D. santomea* backcross males were likewise placed with *D. santomea* parental females. After filming, backcross males were retrieved and saved separately at −80°C for library preparation.

### Behavioral assays

Flies were kept at a 20–22°C, 50% humidity with a constant 12 hour day/night cycle. Male flies were isolated upon eclosion using a light application of CO_2_, aged to 3–5 days and aspirated into plexiglas courtship chambers with a hemispherical well 15 mm in diameter and 3 mm deep. Virgin females were handled similarly, except that they were kept in groups of up to 50 flies. All movies are of single male/female con-specific pairs unless stated otherwise. F1 hybrid males were placed with *D. santomea* virgin females. *D. yakuba*, *D. santomea*, Fl hybrid and backcross flies were filmed 15 minutes each in the first 4 hours after artificial dawn. *D. simulans*, *D. sechellia*, *D. mauritiana*, *D. erecta*, and *D. teissieri* were handled like *D. yakuba* and *D. santomea*, except that courting pairs were filmed for 30 minutes and filming was not restricted to the morning. All movies were shot on a JVC color video camera TK-C1481BEG mounted on a Leica Z6 APO zoom system. Video capture was performed using Pinnacle Video Capture 1.0.1 software (Elgato systems) set at 640×480 pixels, 25 frames per second. High speed video of *D. santomea* males rowing was captured on a Phantom v210 high speed camera (Vision Research) at 640×480 pixels, 1949 frames per second.

### Quantification and analysis of wing rowing

Video analysis was performed using Annotation 1.0 as well as a custom application, wr10.5, which eliminated video in which both flies were running and which was used to manually record wing rows (available on request). Males for which the courtship index (time spent courting divided by total time, abbreviated CI) [10] was less than 0.25 were dropped from the analysis, whereas the remaining males were scored for the number of wing rows, which was divided by the total time (in seconds) that the male spent courting. The phenotype data used for QTL mapping is available as Files S6 (*D. santomea* backcross) and S7 (*D. yakuba* backcross). Rowing frequencies for all wild type species are the average of 5 males from each species, except for *D. yakuba* and *D. santomea*, where rowing frequencies were calculated for ≥20 individuals per line or hybrid genotype. Statistical analysis and jitterplots of wild type *D. yakuba* and *D. santomea* lines, as well as F1 hybrid wing rowing data, was done in R (http://www.r-project.org).

### Library preparation

We estimated chromosome ancestry (“genotypes”) for 192 *D. santomea* backcross progeny and 192 *D. yakuba* backcross progeny with a single Multiplexed Shotgun Genotyping library using 384 barcoded adaptors [13]. In brief, 384 individual gDNA preparations, 192 from each backcross, were restriction digested with MseI and each sample of restricted DNA was ligated to a different barcoded adaptor sequence. All 384 samples were pooled, ethanol precipitated, phenol:chloroform extracted, and purified with the Agencourt AMPure PCR purification kit. The pooled sample was then run on a 1.8% agarose, 0.2% GTG gel, and the 250–350 bp band was extracted and purified using the QIAquick Gel Extraction Kit (Qiagen). The library was amplified (8 cycles) using Phusion Taq (Thermo Scientific) and FC1 and FC2 primers. The PCR product was purified using the Agencourt AMPure PCR purification kit, quantified by qPCR and sequenced in a single lane of an Illumina HiSeq.

### Updating parental genomes

We updated the published *D. yakuba* genome [12] with data from a single lane of HiSeq data each from gDNA libraries made from the parental strains of *D. yakuba* and *D. santomea*. We examined genomewide linkage patterns between markers (Fig. S1A) and identified several regions that did not display strong linkage to immediately adjacent markers in the published *D. yakuba* genome (Table S1). The linkage data implied that two regions of the published *D. yakuba* genome were incorrectly oriented along the second chromosome and that one of these regions was misplaced (Fig. S1A). In addition, multiple small regions on all three chromosomes did not display strong linkage to any other markers genomewide and were masked (Table S1). A custom python script was used to invert, reassemble and mask these regions and is available on request. Repeating the MSG analysis with these re-organized genomes illustrated that these changes had resolved major misassembly issues with the *D. yakuba* genome (Fig. S1B). These re-organized and masked genomes were therefore used for further analysis.

### Parsing data and HMM estimates of ancestry

We used the MSG software pipeline to perfom data parsing and ancestry estimation [13]. Illumina sequencing reads containing data for the backcross progeny were parsed into individual files (see File S1 for the number of reads for each individual). These parsed files were then split into two groups, corresponding to the two different backcrosses, and analyzed separately. Reads were mapped to the parental genomes and ancestries of chromosome regions were estimated with an HMM which had the following priors: the probability distribution for homozygote parent 1:heterozygote:homozygote parent 2 was 0∶0.5∶0.5; the probability that backcross progeny do not contain alleles in reference genomes was 0.1 for both reference genomes; the recombination rate per chromosome was 0.1 (File S2 for the *D. santomea* backcross, File S3 for the *D. yakuba* backcross).

Because the number of positions at which ancestry is estimated by MSG is normally several orders of magnitude greater than the number of recombination breakpoints present in the entire data set, the complete dataset contains many positions with redundant information. Therefore, prior to importing the ancestry data into R/qtl [35], we thinned the dataset to include only neighboring markers with a conditional probability that differed by at least 0.1. The markers at boths ends of a stretch of similar ancestry were retained. The missing data at the ends of most chromosomes in most individuals were then replaced with the prior estimate of ancestry, which, for backcrosses, is 0.5 genomewide (File S4 for the *D. santomea* backcross, File S5 for the *D. yakuba* backcross). These procedures were performed with a custom Python script (https://github.com/dstern/pull_thin).

### QTL analysis

All QTL analysis was performed with R/qtl [35] in R (http://www.r-project.org) in a MacOS environment. The ancestry data were imported into R/qtl with a custom script (https://github.com/dstern/read_cross_msg) to allow direct importation of the conditional probabilities estimated by the HMM of MSG into R/qtl. Genome scans were performed first with a single QTL model using Haley-Knott regression [34] using the scanone function of R/qtl for two measures of wing rowing: wing rows per second of active courtship and wing rows per second of active courtship when the females were stationary. Signficance of QTL peaks was determined by performing 1000 permutations of the data. Because the single-QTL scan revealed QTL on multiple chromosomes in the *D. santomea* backcross and because there appeared to be multiple QTL peaks on the second chromosome in both backcrosses, we built multiple-QTL models to examine the evidence for multiple QTL using the makeqtl, fitqtl, and refineqtl functions of R/qtl. We explored evidence for epistasis between multiple QTL using the addint function of R/qtl. We further examined genomewide evidence for epistasis using the scantwo function of R/qtl.

## Supporting Information

Figure S1 **Rearrangements of the** ***D. yakuba*** **genome based on recombination in the backcross progeny.** (A) The above and below diagonals illustrate the LOD of linkage between markers genome-wide estimated from the *D. santomea* backcross MSG data. Markers on chromosomes 2, 3, and X are illustrated. High LOD is red, low LOD is blue. Multiple regions displayed low LOD between physically continuous markers. (C, D) Two regions on chromosome 2 were estimated from the linkage data to be mis-assemblies resulting from inversion in place (C) and inversion and misplacement (D). Multiple other regions displayed inconsistent patterns of linkage of contiguous markers, including one region on chromosome 3 (E) and one region on chromosome X (F). These regions may be mis-assemblies or they may reflect an artifact of mapping short reads, perhaps resulting from mis-mapping in regions of repetitive DNA. In either case, these regions provide ancestry information that is inconsistent with flanking regions. We therefore chose to mask these regions. (B) Masking these regions and rearranging the two inverted regions resulted in a more consistent pattern of genetic linkage between contiguous markers.
(TIF)

Movie S1 **A** ***D. santomea*** **male rowing in slow motion.** A single *D. santomea* pair of flies was filmed on a Phantom v210 high speed camera (Vision Research) at 1949 frames per second. The video was cropped to the flies and the rowing event and playback adjusted to slow it down 10 fold.
(MOV)

Movie S2 ***D. santomea*** **male rowing while shaking his wings and circling the female.**
(MOV)

Movie S3 **Examples of wing rowing from species in the** ***melanogaster*** **subgroub, and of the slow row-like wing extension in** ***D. erecta*** **.** Several typical rowing examples are given for each species, and the species names are indicated in the text overlay in the movie.
(MOV)

Movie S4 **Rowing and singing.** A *D. santomea* male rowing his wings interspersed with bouts of singing.
(MOV)

Table S1 **Chromosome regions of the original** ***D. yakuba*** **genome that were rearranged or masked prior to MSG analysis.** A list of the coordinates of the regions in the published *D. yakuba* genome that were masked, inverted or moved to generate the revised genome used in the QTL analysis.
(DOCX)

Table S2 **Multiple QTL models.** LOD scores for various multiple QTL models resultant from a two-dimensional genome scan of the *D. santomea* backcross data using the scantwo function in R/qtl to perform Haley-Knott regression on multipoint genotype probabilities. The first column lists the chromosome and marker coordinates for the top-LOD scores for each chromosome pair. The remaining columns list 5 different LOD scores from each of the permution replicates. In order from left to right: Full, the maximum LOD score for the full model with interactions allowed; Two QTL, the difference between the Full LOD and the maximum single-QTL LOD for the chromosome pair; Interaction, the difference between the maximum Full and Full Additive LODs; Full Additive, maximum LOD score for two QTLs with only additive interactions allowed; Two Additive, the difference in LODs between the Full Additive model and the maximum single QTL model for the chromosome pair.
(DOCX)

File S1 **Backcross reads.** This file contains the number of HiSeq reads corresponding to each backcross fly after parsing.
(TXT)

File S2 ***D. santomea*** **backcross genotype data.** This file contains the genotype marker data for the *D. santomea* backcross based on the assignment of individual reads to either parent genome.
(CSV)

File S3 ***D. yakuba*** **backcross genotype data.** This file contains the genotype marker data for the *D. yakuba* backcross based on the assignment of individual reads to either parent genome.
(CSV)

File S4 ***D. santomea*** **backcross genotype pulled thinned.** This file contains ancestry estimates for the *D. santomea* backcross after application of the Hidden Markov Model and thinning to include only neighboring markers whose conditional probability differed by at least 0.1.
(CSV)

File S5 ***D. yakuba*** **backcross genotype pulled thinned.** This file contains ancestry estimates for the *D. yakuba* backcross after application of the Hidden Markov Model and thinning to include only neighboring markers whose conditional probability differed by at least 0.1.
(CSV)

File S6 ***D. santomea*** **backcross phenotype data.** This file contains the phenotype data for the *D. santomea* backcross population. Columns, from left to right: movie and barcode identification, wing rows pers seconds courtship, wing rows per seconds the female was stationary during courtship, sex of fly.
(CSV)

File S7 ***D. yakuba*** **backcross phenotype.** This file contains the phenotype data for the *D. yakuba* backcross population. Columns, from left to right: movie and barcode identification, wing rows pers seconds courtship, wing rows per seconds the female was stationary during courtship, sex of fly.
(CSV)
