# Supplementary figures and images for: Evolution of Multiple Additive Loci Caused Divergence between Drosophila yakuba and D. santomea in Wing Rowing during Male Courtship
Source: PLoS One. 2012 Aug 30;7(8):e43888. doi: 10.1371/journal.pone.0043888 (PMC3431401; doi:10.1371/journal.pone.0043888)

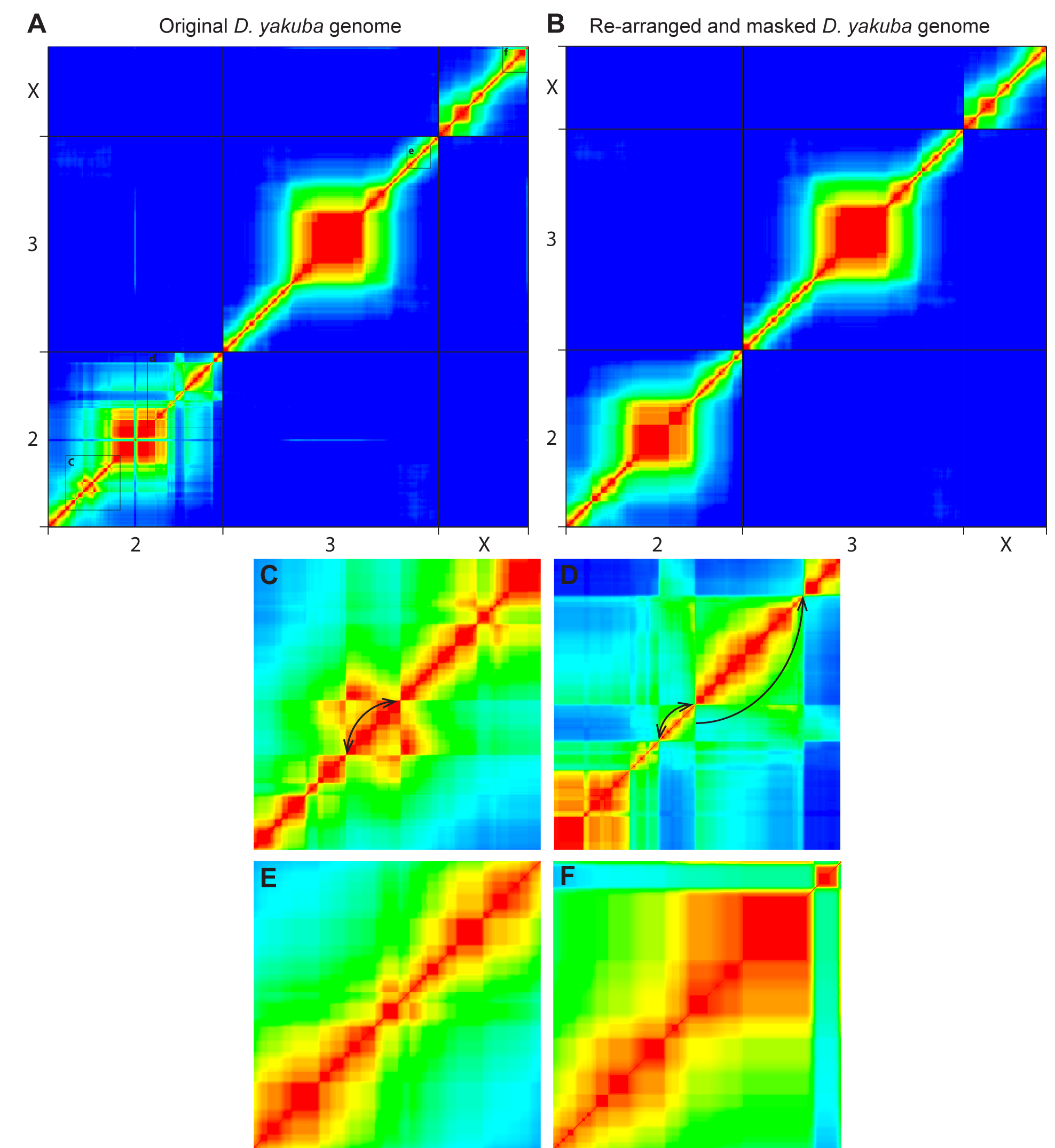

Supplement: Figure S1 — Rearrangements of the D. yakuba genome based on recombination in the backcross progeny. (A) The above and below diagonals illustrate the LOD of linkage between markers genome-wide estimated from the D. santomea backcross MSG data. Markers on chromosomes 2, 3, and X are illustrated. High LOD is red, low LOD is blue. Multiple regions displayed low LOD between physically continuous markers. (C, D) Two regions on chromosome 2 were estimated from the linkage data to be mis-assemblies resulting from inversion in place (C) and inversion and misplacement (D). Multiple other regions displayed inconsistent patterns of linkage of contiguous markers, including one region on chromosome 3 (E) and one region on chromosome X (F). These regions may be mis-assemblies or they may reflect an artifact of mapping short reads, perhaps resulting from mis-mapping in regions of repetitive DNA. In either case, these regions provide ancestry information that is inconsistent with flanking regions. We therefore chose to mask these regions. (B) Masking these regions and rearranging the two inverted regions resulted in a more consistent pattern of genetic linkage between contiguous markers. (TIF) [file pone.0043888.s001.tif]
