# Supplementary material for: Evolution of Multiple Additive Loci Caused Divergence between Drosophila yakuba and D. santomea in Wing Rowing during Male Courtship
Source: PLoS One. 2012 Aug 30;7(8):e43888. doi: 10.1371/journal.pone.0043888 (PMC3431401; doi:10.1371/journal.pone.0043888)
Supplement: Table S1 — Chromosome regions of the original D. yakuba genome that were rearranged or masked prior to MSG analysis. A list of the coordinates of the regions in the published D. yakuba genome that were masked, inverted or moved to generate the revised genome used in the QTL analysis. (DOCX) [file pone.0043888.s006.docx]

Table S1. Chromosome regions of the original *D. yakuba* genome that were rearranged or masked prior to MSG analysis.

| ^[[1]](#footnote-1)^Chromosome^*^ | ^[[2]](#footnote-2)^Start^†^ | Stop^†^ | ^[[3]](#footnote-3)^Masked^‡^ | ^[[4]](#footnote-4)^Inverted^§^ | Moved To^†^ |
| --- | --- | --- | --- | --- | --- |
| 2 | 1972482 | 2264257 | Yes |  |  |
| 2 | 6351304 | 7392780 | Yes |  |  |
| 2 | 10597789 | 11122228 | Yes |  |  |
| 2 | 14526636 | 15540523 | Yes |  |  |
| 2 | 20988046 | 21999891 | Yes |  |  |
| 2 | 26265701 | 26683986 | Yes |  |  |
| 2 | 26684004 | 27352929 | Yes |  |  |
| 2 | 29255778 | 29695959 | Yes |  |  |
| 2 | 30265066 | 30545009 | Yes |  |  |
| 2 | 39211389 | 39695129 | Yes |  |  |
| 2 | 40066768 | 40540071 | Yes |  |  |
| 2 | 8595311 | 11122687 |  | Yes |  |
| 2 | 31108624 | 33485515 |  | Yes | 40462161 |
| 3 | 10849160 | 11358200 | Yes |  |  |
| 3 | 15550215 | 16076720 | Yes |  |  |
| 3 | 40124281 | 40349801 | Yes |  |  |
| 3 | 42230532 | 42965291 | Yes |  |  |
| 3 | 47400361 | 47715389 | Yes |  |  |
| X | 20394985 | 21750564 | Yes |  |  |

* “Chromosomes” 2L and 2R from the published genome were concatenated to make chromosome 2, and “chromosomes” 3L and 3R were concatenated to make chromosome 3.

† In bp.

‡ Bases in these regions were replaced with Ns.

§ The reverse complement sequence was generated.

1. [↑](#footnote-ref-1)
2. [↑](#footnote-ref-2)
3. [↑](#footnote-ref-3)
4. [↑](#footnote-ref-4)
