# Supplementary material for: Evolution of Multiple Additive Loci Caused Divergence between Drosophila yakuba and D. santomea in Wing Rowing during Male Courtship
Source: PLoS One. 2012 Aug 30;7(8):e43888. doi: 10.1371/journal.pone.0043888 (PMC3431401; doi:10.1371/journal.pone.0043888)
Supplement: Table S2 — Multiple QTL models. LOD scores for various multiple QTL models resultant from a two-dimensional genome scan of the D. santomea backcross data using the scantwo function in R/qtl to perform Haley-Knott regression on multipoint genotype probabilities. The first column lists the chromosome and marker coordinates for the top-LOD scores for each chromosome pair. The remaining columns list 5 different LOD scores from each of the permution replicates. In order from left to right: Full, the maximum LOD score for the full model with interactions allowed; Two QTL, the difference between the Full LOD and the maximum single-QTL LOD for the chromosome pair; Interaction, the difference between the maximum Full and Full Additive LODs; Full Additive, maximum LOD score for two QTLs with only additive interactions allowed; Two Additive, the difference in LODs between the Full Additive model and the maximum single QTL model for the chromosome pair. (DOCX) [file pone.0043888.s007.docx]

Supplementary Table 2. Results of two-QTL scan for *D. santomea* backcross.

|  | **LOD** | | | | |
| --- | --- | --- | --- | --- | --- |
| **Chromosomes** | **Full** | **Two QTL** | **Interaction** | **Full Additive** | **Two Additive** |
| chr2.15687032 : | 13.0*** | 3.3 | 0.28 | 12.7*** | 3.03* |
| chr2.43343589 |  |  |  |  |  |
| chr2.15806943 : | 14.2*** | 4.5** | 0.12 | 14.0*** | 4.40*** |
| chr3. 46460168 |  |  |  |  |  |
| chr2.15687032 : | 10.7*** | 1 | 0.79 | 9.9*** | 0.25 |
| chr4.17524 |  |  |  |  |  |
| chr2.15687032 : | 11.4*** | 1.7 | 0.67 | 10.7*** | 1.05 |
| chrX.21761345 |  |  |  |  |  |
| chr3.46990315 : | 6.7*** | 2 | 0.18 | 6.5*** | 1.79 |
| chr3.51479632 |  |  |  |  |  |
| chr3.46990315 : | 5.5** | 0.8 | 0.09 | 5.4** | 0.69 |
| chr4.1369022 |  |  |  |  |  |
| chr3. 46990315 : | 6.4** | 1.7 | 1.07 | 5.3** | 0.6 |
| chrX. 20299987 |  |  |  |  |  |
| chr4.1332793 : | 2.5 | 2 | 0.13 | 2.4 | 1.82 |
| chr4. 1353389 |  |  |  |  |  |
| chr4.1100 : | 1.8 | 1.1 | 0.44 | 1.3 | 0.64 |
| chrX. 13576477 |  |  |  |  |  |
| chrX.14236352 : | 3.3 | 2.6 | 1.43 | 1.9 | 1.16 |
| chrX.14238513 |  |  |  |  |  |

*P < 0.05

** P < 0.01

*** P < 0.001
